# Very small embryonic-like stem cells (VSELs) detected in azoospermic testicular biopsies of adult survivors of childhood cancer

**DOI:** 10.1186/s12958-015-0121-1

**Published:** 2015-11-09

**Authors:** Purna Kurkure, Maya Prasad, Vandana Dhamankar, Ganesh Bakshi

**Affiliations:** ACT Clinic, Department of Pediatric Oncology, Dr E Borges Road, Parel, Mumbai, 400012 India; Division of Uro-oncology, Tata Memorial Hospital, Dr E Borges Road, Parel, Mumbai, 400012 India

**Keywords:** Cancer, Oncotherapy, Azoospermia, VSELs, Spermatogenesis, Stem cells

## Abstract

**Background:**

Infertility is a known side-effect of oncotherapy in cancer survivors, and often compromises the quality of life. The present study was undertaken to detect very small embryonic-like stem cells (VSELs) in testicular biopsies from young adult survivors of childhood cancer who had azoospermia. VSELs have been earlier reported in human and mouse testes. They resist busulphan treatment in mice and potentially restore spermatogenesis when the somatic niche is restored by transplanting Sertoli or mesenchymal cells. VSELs also have the potential to differentiate into sperm in vitro.

**Methods:**

The study had clearance from Institutional review board (IRB). Seven azoospermic survivors of childhood cancer were included in the study after obtaining their informed consent. Semen analysis was done to confirm azoospermia prior to inclusion in the study. Testicular biopsies were performed at the Uro-oncology Unit of the hospital and then used for various studies to detect VSELs.

**Results:**

Hematoxylin and Eosin stained tubular sections confirmed azoospermia and smears revealed the presence of very small, spherical VSELs with high nucleo-cytoplasmic ratio, in addition to the Sertoli cells. Immuno-localization studies on testicular smears showed that the VSELs were CD133+/CD45-/LIN-, expressed nuclear OCT-4, STELLA and cell surface SSEA-4. Pluripotent transcripts Oct-4A, Nanog and Sox-2 were detected in azoospermic samples whereas marked reduction was observed in germ cell markers Oct-4 and Boule.

**Conclusions:**

The present study demonstrates the presence of pluripotent VSELs in the testicular biopsy of azoospermic adult survivors of childhood cancer. It is likely that these persisting VSELs can restore spermatogenesis as demonstrated in mice studies. Therefore, pilot studies need to be undertaken using autologous mesenchymal cells with a hope to restore testicular function and fertility in cancer survivors. The results of this study assume a great significance in the current era, where cryopreservation of testicular tissue in young pre-pubertal boys for restoring spermatogenesis in adulthood is still in experimental stages.

## Background

With recent advances in the field of oncotherapy, more than 80 % pediatric patients survive cancer [1]. This has resulted in an increasing focus on the late effects of therapy and quality of life in the growing population of childhood cancer survivors. Approximately 2 of every 3 childhood cancer survivors will experience at least one late effect, and 40 % may develop a ‘severe, disabling, or life-threatening condition’ 30 years after cancer diagnosis [2].

Late effects on the reproductive system can be manifold. Chemotherapy, surgery and radiotherapy can affect either the hypothalamo-pituitary axis or the gonads themselves, and can lead to infertility and other side effects on the reproductive system. Several studies have found that infertility is a common late effect of treatment in adult male survivors of childhood cancer [3–7]. A large study found that cancer survivors have a lower chance of siring a pregnancy than their siblings (hazard ratio 0.56) [3]. The treatment factors commonly implicated in male gonadal dysfunction include cumulative alkylating agent (cyclophosphamide and procarbazine) dose, radiation to testes, pelvis/abdomen and hypothalamus/pituitary and orchidectomy [3, 4, 8–10].

Presently, sperm banking is the only widely established and successful fertility preservation method for males, but is technically challenging and even not possible at times in children and teenagers. Importantly, the diagnosis of a malignancy is devastating to the child’s family and treatment is usually initiated urgently. Discussions regarding late effects including infertility tend to take a backseat, especially in resource poor settings. The only available options today in pre pubertal boys is testicular tissue cryopreservation as tubules or germ cells and later transplantation or testis xenografting to obtain mature sperm for assisted reproduction. These are yet experimental approaches [11, 12]. Due to the above reasons, fertility preservation is often a low priority in Paediatric Oncology centers. In many instances, adult survivors of childhood cancer become aware of this late effect only when confronted with infertility and at this stage treatment options are limited and extremely disappointing. They undergo severe psychosocial trauma and often wish that they had more information and options regarding their fertility, and that they had undergone fertility preservation prior to cancer treatment.

Besides sperm banking and testicular biopsy for future use, research on pluripotent stem cells has provided a ray of hope as these stem cells have potential to differentiate into ‘synthetic’ gametes. Several groups across the world have reported differentiation of pluripotent embryonic stem (ES) cells and induced pluripotent stem (iPS) cells into germ cells. However, these approaches remain highly inefficient and are still a distant dream [13]. Besides ES and iPS cells, an additional novel population of pluripotent stem cells termed very small embryonic-like stem cells (VSELs) reported in various adult organs including testis and ovary [14–18] may serve as an alternative candidate to obtain gametes for achieving biological parenthood [19]. These stem cells are relatively quiescent in nature and thus have been reported to survive total body irradiation in mouse bone marrow [20] and after busulphan and cyclophosphamide treatment in mouse testis [21] and ovary [22]. VSELs are located in the basal layer of seminiferous epithelium in the testis and in the ovary surface epithelium. They are pluripotent cells believed to be the primordial germ cells which persist throughout life albeit in few numbers [23] and get mobilized under disease conditions to regenerate and bring about homeostasis [24]. VSELs indeed serve as a backup pool of stem cells which occasionally self-renew themselves and also give rise to the progenitors by undergoing asymmetric cell divisions. The progenitors including spermatogonial stem cells (SSCs) in testis and ovary germ stem cells (OGSCs) in ovary divide by undergoing rapid symmetric cell divisions followed by further differentiation and meiosis to form the gametes. Existence of similar two populations including ‘dormant’ and ‘restless’ stem cells has been also reported in gut epithelium, hair follicle, bone marrow and skin [25–27].

Anand et al. [21] demonstrated that the testicular VSELs which survive busulphan treatment in mice are capable of restoring spermatogenesis and the caudal sperm fertilized oocytes in vitro resulting in early embryonic development. This was achieved by providing a healthy somatic niche to the surviving VSELs by way of inter-tubular transplanting Sertoli or mesenchymal cells. The transplanted cells provided a source of growth factors and cytokines required for VSELs to undergo spermatogenesis. Similarly the surviving VSELs in chemoablated mice ovaries also initiated differentiation into oocytes [22]. Anand et al. [28] have also recently studied the potential of VSELs surviving in chemoablated testis to undergo spermatogenesis in vitro. Since VSELs have been reported in adult human testicular tissue earlier [14] the present study was undertaken to investigate whether the VSELs survive in otherwise azoospermic testicular biopsies of adult survivors of childhood cancer. Presence of VSELs in azoospermic testicular biopsies open up an altogether novel approach to restore fertility with an aim to achieve biological parenthood and thus indirectly help improve quality life of cancer survivors.

## Methods

Approval was obtained from the IRB of Tata Memorial Hospital, Mumbai to collect testicular biopsy from male survivors of childhood cancer and one normal testicular sample was obtained from a patient undergoing orchidectomy as part of treatment for prostate cancer as normal control.

### Testicular biopsy collection

Seven adult survivors of various types of childhood cancers were evaluated for infertility in After Completion of Therapy (ACT) clinic for long term survivors of childhood cancers using semen analysis, circulating follicular stimulating hormone (FSH) and testosterone levels prior to being enrolled for the study. Once azoospermic status was confirmed, the participants were subjected to a testicular biopsy under sterile conditions. In brief, 10–12 pieces of testicular biopsy, each measuring 1–2 mm^3^ were collected sites from a single testis under local anesthesia. The procedure lasted about 10–15 min. Normal testicular tissue was also collected from a patient undergoing orchidectomy as part of treatment towards prostate cancer after appropriate informed consent. The specimens were collected in sterile DMEM F12 medium (Invitrogen, USA) with antibiotics on ice and were processed appropriately for histology, smear preparation and for RNA extraction.

### Tissue processing

A part of human testicular biopsy was fixed in 10 % neutral buffer formalin for histological studies. 5 μm thick sections of paraffin embedded tubules were prepared and stained with Hematoxylin and eosin (H & E) to study the tissue histo-architecture. Testicular cell smears were prepared by fine chopping of tissue and filtration through a 40 μm filter. The smears were then fixed with 4 % paraformaldehyde and stored at 4 °C till further use. A portion of tissue was fixed in Trizol (Invitrogen) for RNA isolation and stored at -80 °C till further processing.

### Immuno localization studies

The cell smears were used to immuno-localize pluripotent markers (OCT-4; SSEA-4), primordial germ cell marker (STELLA) and for VSELs specific marker (CD133). The cells were permeabilized using 0.3 % Triton-X 100 for 5 min following washes with 0.5 % bovine serum albumin (BSA). The permeabilization step was omitted for cell surface markers. Blocking was done with 3 % BSA solution for 1 h. Smears were incubated overnight with primary antibodies namely OCT-4 (Abcam, UK), SSEA-4 (Millipore, USA), STELLA (Millipore), CD133 (Miltenyi, USA) at 4 °C. For co-localization study, directly tagged CD133-APC (Miltenyi, USA), LIN - FITC (BD) and CD45-PE (BD) were used. After washing with 0.5 % BSA, the smears were blocked with 1 % BSA and 5 % normal goat serum for 1 h followed by incubation with antibodies for 2 h at room temperature. The cells were then washed thrice with wash buffer and counterstained with DAPI (4',6-diamidino-2-phenylindole dihydrochloride) for 15 min. All images were captured by laser scanning confocal microscope (Carl Zeiss, Germany) at 63 X magnification.

### Quantitative RT-PCR

Extracted RNA was treated with DNase I (Amersham Biosciences, USA) to remove any genomic DNA contamination. First-strand cDNA was synthesized using the iScript cDNA synthesis Kit (Bio-Rad, USA) according to the manufacturer’s instructions. 1 μg of total RNA was incubated with 5x iScript reaction mix and reverse transcriptase mix. The reaction was carried out in G-STORM thermocycler (Gene Technologies, UK). The reaction mix was first incubated at 25 °C for 5 min, then at 42 °C for 30 min and finally at 85 °C for 5 min. The expression levels of various gene transcripts, namely total Oct-4A, Oct-4B, Nanog, Sox-2, Boule, and TP1 gene transcripts were studied using 18 s as housekeeping gene, and were estimated by CFX96 real-time PCR system (Bio-Rad Laboratories, USA) using SYBR Green chemistry (Bio-Rad). The primers used in the study are mentioned in Table 1. The amplification conditions were: initial denaturation at 94 °C for 3 min followed by 40 cycles comprising of denaturation at 94 °C for 10 s, primer annealing for 20 s, and extension at 72 °C for 30 s followed by melt curve analysis step from 55 °C to 95 °C. The fluorescence emitted at each cycle was collected during the extension step of each cycle. The homogeneity of the PCR amplicons was verified by running the products on 2 % agarose gels and also by studying the melt curve. All PCR amplifications were carried out in duplicate. Mean Ct values generated in each experiment using the CFX Manager software (Bio-Rad) were used to calculate the mRNA expression levels. Since ΔCt is inversely proportional to relative mRNA expression levels, the levels were calculated manually by the ΔCt method.

**Table 1:** Primer sequences for various transcripts used in the study

Primer		Sequence	Annealing
Oct-4A	F R	CTCCTGGAGGGCCAGGAATC CCACATCGGCCTGTGTATAT	62 °C
Oct-4B	F R	GCTTCTGAGACATGATGCTCTTCC CTCACTCAAGTATCACCCCCAGT	62 °C
Nanog	F R	AGTCCCAAAGGCAAACAACCCACTTC TGCTGGAGGCTGAGGTATTTCTGTCTC	62 °C
Sox-2	F R	GGGGGAAAGTAGTTTGCTGCCTCT TGCCGCCGCCGATGATTGTT	62 °C
Boule	F R	CAGTGCCGCAACTTGCT GGCACTTGTTGGGTTATTCAAAGG	55 °C
18S	F R	GGAGAGGGAGCCTGAGAAAC CCTCCAATGGATCCTCGTTA	61 °C

## Results

The clinical and previous treatment details of the seven adult survivors of childhood cancer included in the study are as described in Table 2. Initial screening by semen analysis and hormone assays confirmed that all participants were azoospermic and had normal to raised FSH and testosterone levels. All seven survivors agreed to participate in the study with a strong desire to attain biological parenthood, although they were informed about the basic nature of the present study.

**Table 2:** Details of the Human Participants Included in the Study

No	Current Age of Cancer Survivor	Age at Diagnosis of Cancer	Type of Cancer; Treatment Regimen given to Treat Cancer (with alkylating agent doses)	Semen Analysis	FSH	Testo-sterone
1	35 years	12 years	Hodgkin’s Lymphoma; CT + RT CT : Cyclophosphamide-9.6gm/m^2^ RT to bilateral neck 40Gy/20#	Azoospermia	-	-
2	35 years	12.6 years	Lymphoblastic lymphoma;CT+ RT CT : Cyclophosphamide 22.4gm/m^2^ RT : Cranial 18Gy/9# Mediastinum 40 Gy/20#	Azoospermia	-	-
3	30 year	5 years at first diagnosis 27 years at recurrence	Non Hodgkin’s lymphoma with recurrence/ transformation to Diffuse Large B cell Lymphoma Surgery + CT+ RT Surgery-Right Orchiectomy CT: Cyclophosphamide 16gm/m^2^ RT: Cranial 18Gy/9# CT alone Cyclophosphamide-5.5 gm/m^2^	Azoospermia	WNL	WNL
4	35 years	8 years	Wilms tumor : Surgery + CT+ RT Surgery-Left nephrectomy CT: Cyclophosphamide-10.8 gm/m^2^, RT to left renal fossa 20 Gy/7#	Azoospermia	Elevated	WNL
5	28 years	7 years	Hodgkin Lymphoma; CT + RT CT : Cyclophosphamide- 9.3 gm/m^2^; Procarbazine-10 gm/m^2^ RT to bilateral neck 36 Gy/18#	Azoospermia	Elevated	WNL
6	23 years	13 years	Non Hodgkin lymphoma;CT + RT CT :Cyclophosphamide 20 g/m^2^ RT: Cranial 19.8Gy/11#	Azoospermia	Elevated	Elevated
7	25 years	10 year	Non Hodgkin lymphoma; CT + RT CT : Cyclophosphamide 19gm/m^2^ RT: Cranial 19.8Gy/11#	Azoospermia	Elevated	Elevated

*CT – chemotherapy; RT- Radiotherapy; Gy –gray; WNL- Within normal range*

**Fig. 1:**
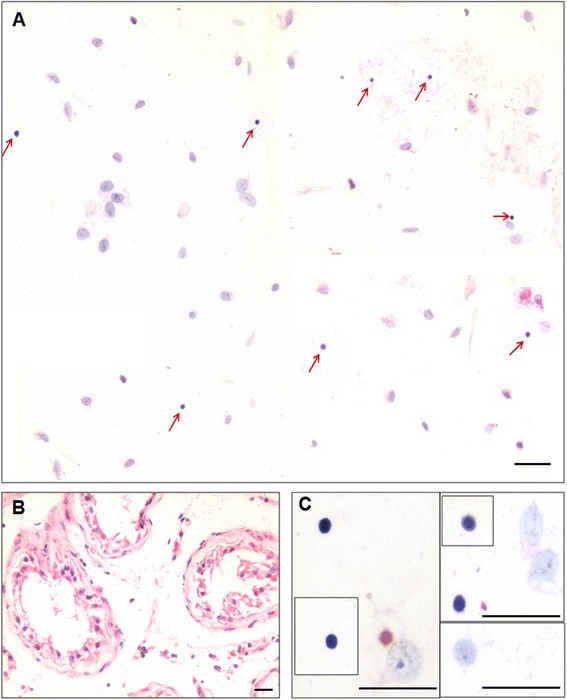
VSELs in testicular biopsy of azoospermic men survivors of childhood cancer. **a** Testicular smear showing the presence of two types of cells including Sertoli cells (with abundant cytoplasm, pale stained nuclei and prominent nucleolus) and spherical, small sized VSELs with dark stained nuclei (arrows). Please note that several fields have been combined to make this composite. (**b**) H & E stained testicular section showing empty tubules completely devoid of germ cells and sperm (**c**) Various representative fields showing Sertoli cells and VSELs at higher magnification. Scale: 20 μm

**Fig. 2:**
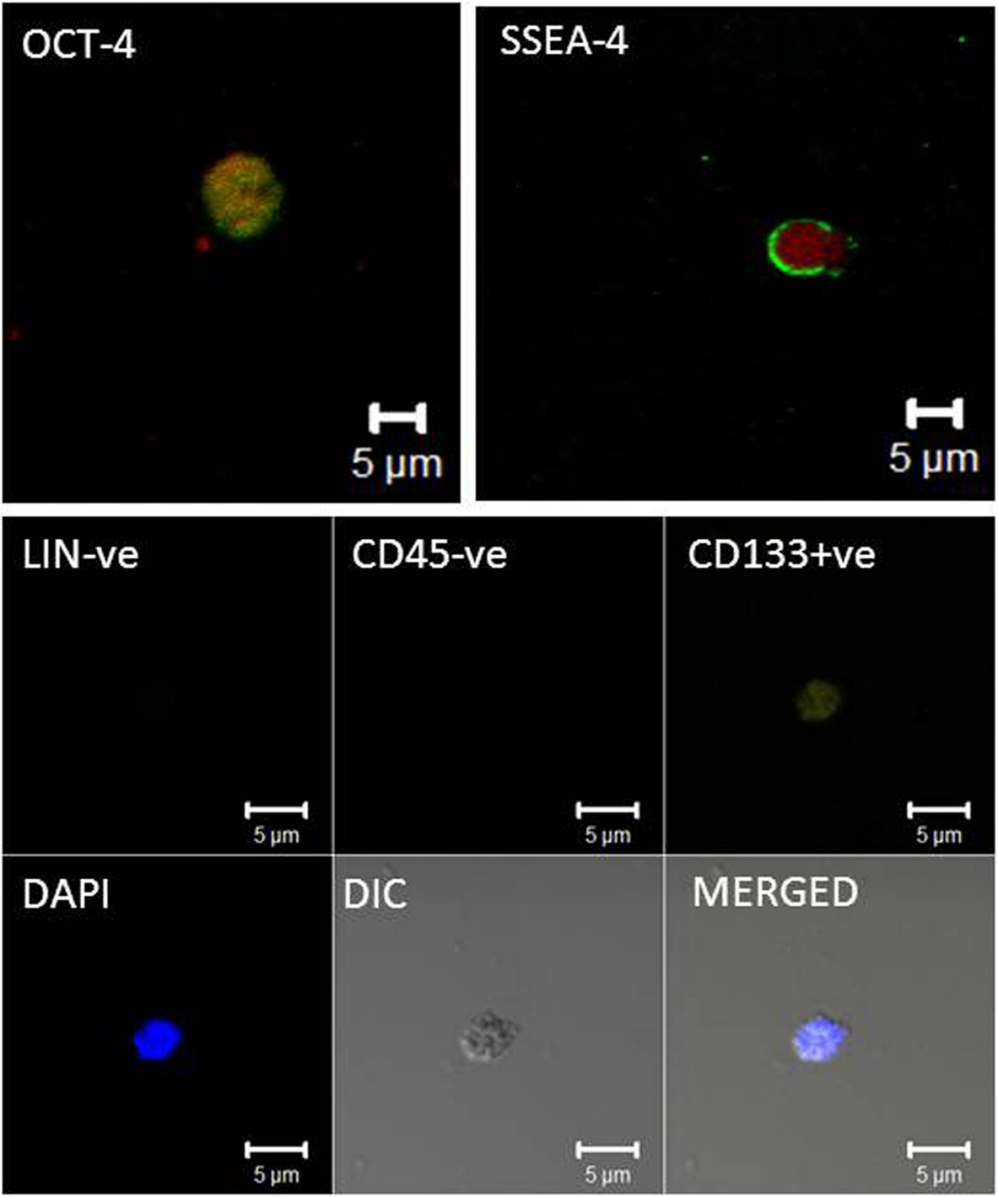
Immuno-localization of nuclear OCT-4 and cell surface SSEA-4 with propidium iodide as counterstain. Lower panel shows a single VSEL stained for LINEAGE and CD45 markers (both negative) but is positive for CD133 and DAPI

**Fig. 3:**
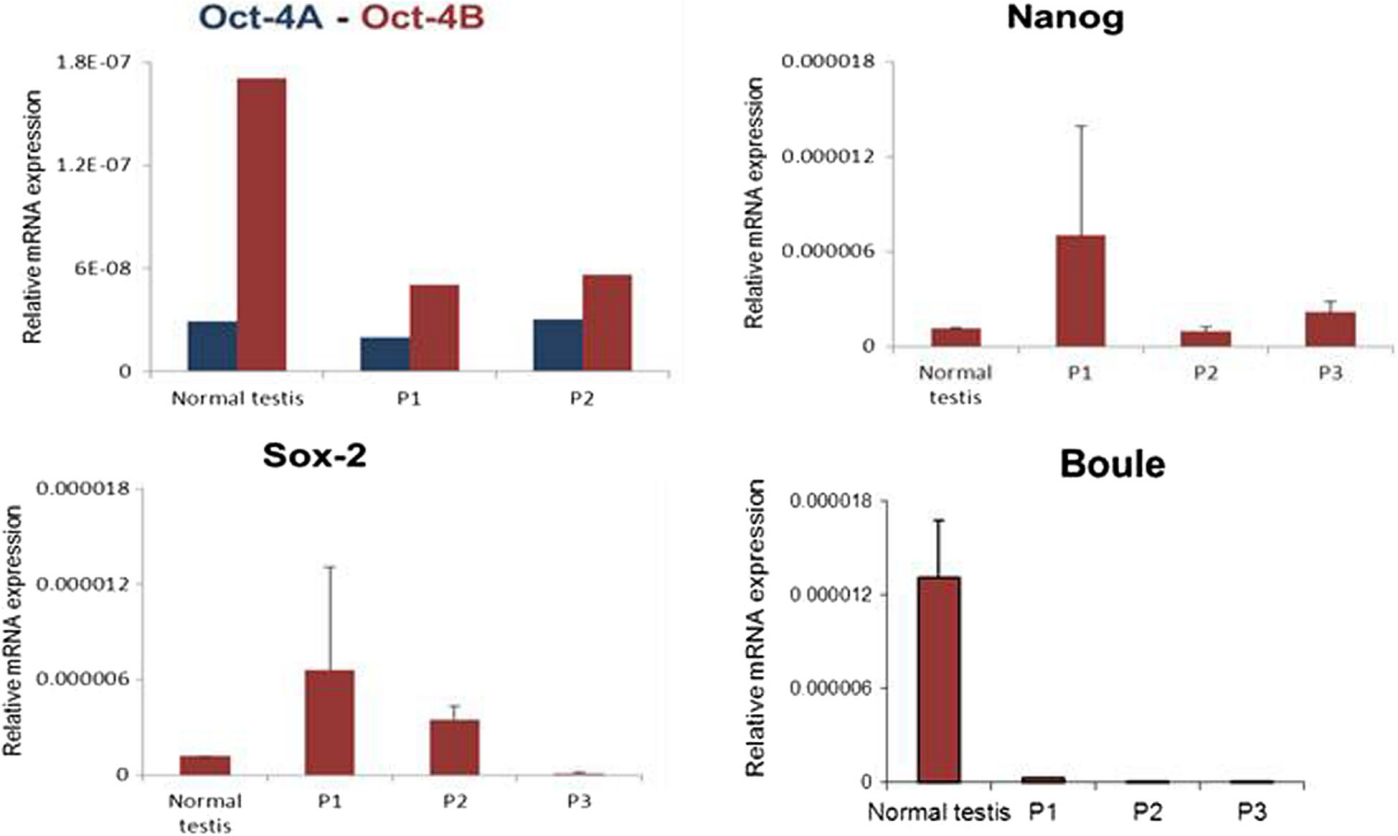
Relative mRNA expression of pluripotent markers (Oct-4A/Oct-4B, Nanog, Sox-2) and germ cell specific marker (Boule) by qRT-PCR in azoospermic testes compared to normal testicular sample. Note that the pluripotent markers persist (unaltered or slightly increased) whereas the germ cell marker was markedly reduced in azoospermic testicular sample. Pluripotent transcripts expression for various patients (P1, P2 and P3) is represented separately as relative expression normalized to 18S RNA. Error bars represent standard deviation between two technical replicates for the same patient sample

The H&E stained testicular sections confirmed the presence of azoospermia as evidenced by the presence of empty tubules with no signs of germ cells and sperm (Fig. 1). The testicular cell smears comprised of Sertoli cells and VSELs. VSELs were identified as small sized, spherical in shape and had a typical dark Hematoxylin stained nucleus and high nucleo-cytoplasmic ratio (Fig. 1). Sertoli cells had abundant cytoplasm and characteristic pale stained nuclei and prominent nucleolus (Fig. 1). Immuno-fluorescence studies were done to characterize VSELs for the expression of pluripotency and VSEL specific markers namely OCT-4 and SSEA-4 (Fig. 2). Also, co-localization studies showed CD133 positive cells were negative for LIN and CD45 markers (Fig. 2). The presence of pluripotent VSELs in the azoospermic testicular biopsy was further confirmed by the differential expression of pluripotent (Oct-4A, Nanog, Sox2) and germ cell (Boule) specific transcripts by qRT-PCR. The azoospermic samples revealed unaltered or slightly increased expression of pluripotent markers (Oct-4A, Nanog and Sox2) (Fig. 3) whereas the germ cell marker Boule was down regulated compared to normal testicular sample. Although Oct-4A levels remained similar to normal testicular sample, Oct-4B was reduced indicating decrease in actively dividing progenitors. Thus, similar to the effect of busulphan treatment in mice [21], VSELs survived post-oncotherapy in humans whereas germ cells and sperm were completely lacking in the azoospermic testicular biopsies.

## Discussion

With improving childhood cancer treatment protocols in past few decades more child hood cancer survivors have been detected with late side effects of such oncotherapy. The two important aspects of this issue are (i) to risk-adapt and optimize treatment protocols in order to reduce the incidence of side effects while maximizing cure and (ii) monitoring and management of late effects of childhood cancer survivors. The bulk of current translational and clinical research in pediatric oncology focuses on the first aspect and children treated on current protocols are anticipated to have fewer and less severe late effects of treatment [29, 30]. The second aspect of managing survivors at high risk of late effects or those already facing late effects is more challenging.

At Tata Memorial Hospital Mumbai, the After Completion of Therapy (*ACT*) Clinic was set up in 1991 for the comprehensive follow- up of childhood cancer survivors. This clinic drew its inspiration from the model of care established at St. Jude Children Research Hospital, USA, and attempts to achieve “cure” in its true perspective [31]. At present there are 1700 survivors (disease free for more than 2 years after cessation of therapy) who are registered in the *ACT* Clinic. Infertility was recognized as a serious late effect in our cohort of male cancer survivors [32]. Premarital fertility counseling is routinely offered to all young adult survivors, which includes discussion about their fertility issues, semen analysis, available options like donor sperm or adoption and most importantly sharing the disease status with prospective spouse.. Since we do not have in-house facilities for Assisted Reproduction Technologies (ART), we refer our infertile survivors to appropriate specialists at other Centres.

*ACT* Clinic database for reproductive outcome in married young adult survivors revealed that 604 young adult survivors (> 18 years of age) were registered of whom 188 were females and 416 were males. Among the female cancer survivors, 89 are lost to follow-up, 21 are married, 1 is divorced and 11 of the unmarried female cancer survivors are on hormone replacement therapy. Fertility outcome available in 13 married females shows that 10 have normal offspring and 3 are currently pregnant. So far, none of the married females have approached us with the concern of infertility. Amongst the 416 male survivors, 180 are lost to follow up. Of the 70 married male survivors, only 30 were able to sire a pregnancy naturally; the rest were found to have either azoospermia or oligospermia when evaluated for complaints of infertility or during premarital counseling. 17 of these infertile survivors underwent assisted reproductive techniques (successful in 6 instances), 3 have adopted and the remaining 20 currently do not have children. All the offspring born to both male and female cancer survivors have been reported to be healthy. Of the 40 azoospermic/ oligospermic survivors, the most common diagnosis was Hodgkin Lymphoma (HL; *n* = 27) followed by Acute Lymphoblastic Leukemia (ALL; *n* = 4), Non Hodgkin Lymphoma (NHL; *n* = 3) and others (*n* = 3) . The median current age of this cohort is 31 years (range 20–53 years), with the median age at diagnosis being 8.5 years (range 3–16 years), and the time since cessation of treatment being 19.5 years (range 5–38 years). 28/40 had received alkylating agents (high dose cyclophosphamide and /or procarbazine), 4 ALL survivors had received cranial radiation and 3 had received abdomino-pelvic radiation. The numbers were too small to derive statistical significance. Seven of these patients were enrolled in this study .The individual clinical details are in Table 2. All had received alkylating agents and/or radiation. The median time between treatment completion and enrolment into the trial was 23 years (median 21–27 years).

We counseled 15 of these cancer survivors and 7 agreed to give a testicular biopsy for the present study. They were highly motivated to participate in the study with a hope that the results may benefit them in near future. This study reveals the presence of VSELs in testicular biopsies collected from otherwise azoospermic adult survivors of childhood cancer. The pluripotent VSELs exhibited characteristic spherical shape, high high nucleo-cytoplasmic ratio, dark stained nuclei and expressed nuclear OCT-4, cell surface SSEA-4 and STELLA. As expected the testicular VSELs were LIN-/CD45-/CD133+. Also the presence of VSELs was confirmed at mRNA level by the expression of pluripotent transcripts Oct-4A, Nanog and Sox2. Absence of germ cells was confirmed by histological studies and lack of amplification of germ cell marker Boule. Due to scarcity of clinical material extensive flow cytometry studies could not be undertaken. Presence of these stem cells in the azoospermic human testicular biopsies raises a lot of hope. Are these the same VSELs which survived oncotherapy during childhood and also whether their functionality is affected by oncotherapy - are difficult questions to answer. VSELs are known to be easily mobilized under stress conditions [24] and they survive because of their quiescent nature. Thus we do not think their functionality will be in anyways compromised due to oncotherapy and will be mobilized from bone marrow if body requires. Thus a pilot study wherein autologus bone marrow/adipose tissue derived mesenchymal cells expanded in a GMP facility are transplanted in azoospermic testis needs to be urgently undertaken with a hope to restore gonadal function from these stem cells.

Given the magnitude of the infertility concern in our young adult survivors of childhood cancer (especially in males), and the limited treatment options in the absence of prior semen cryopreservation in any of the patients, the results of this study have a huge and positive implication. A significant question is whether the surviving VSELs observed in the testicular biopsies of cancer survivors will be able to restore spermatogenesis when the niche cells are transplanted. It was recently reported that inter-tubular random injections of syngenic Sertoli cells or bone marrow derived mesenchymal cells into the chemoablated testes of mice, were able to restore spermatogenesis from persisting VSELs in mice, and that the sperm developed post transplantation were found to be capable of fertilizing mouse eggs [21]. Also, when cells from chemoablated tubules were cultured, the inhibitory factors in vivo were overcome, Sertoli cells provided the niche and the surviving VSELs spontaneously differentiated into sperm [28]. Similar VSELs also exist in adult ovary surface epithelium [15, 17] and survive chemotherapy [22]. Culture of intact ovary or surface epithelial cells isolated from ovary of chemoablated mice showed spontaneous differentiation into oocyte-like structures since the inhibitory factors were overcome in vitro [22]. These studies in mice highlight the potential of VSELs and also the importance of niche to support their function.

The recent editorial in Nature Medicine concludes that for achieving regeneration from the stem cells – niche cannot be ignored [33, 34]. Providing a healthy niche to the testis of these azoospermic men could possibly lead to resumption of spermatogenesis from surviving VSELs. The results in mice are yet to be replicated in humans. Azoospermic adult cancer survivors are potential beneficiaries. Autologus mesenchymal cells from bone marrow/adipose tissue may be expanded in culture and transplanted through inter-tubular route as a simple outpatient procedure. They may act as a source of growth factors and cytokines required for VSELs differentiation into sperm. It is essential to note that the transplanted mesenchymal cells do not differentiate into sperm (although available literature may be confusing) – rather mesenchymal cells facilitate differentiation of endogenous VSELs into sperm. Thus the VSELs that survive oncotherapy in cancer survivors may be targeted to restore fertility in cancer survivors. VSELs and their potential to restore gonadal function will result in a huge paradigm shift in the field of oncofertility, but the results of this study need to be validated by other groups and further studies in humans need to be urgently undertaken. A similar clinical trial has already been registered in the cancer trial registry of US FDA [35]. In children undergoing cancer treatment currently, cryopreservation of testicular tissue could serve as a source of both germ cells and autologous Sertoli cells which are the natural niche for testicular germ cells. The approach of targeting endogenous VSELs to restore testicular function by providing a healthy niche could obviate the need for expensive ART in the future.

## Conclusions

Premature gonadal failure and subsequent infertility are important and permanent late effects in adult survivors of childhood cancers. The demonstration of testicular VSELs which persist post cancer treatment and regain their functionality in the presence of a healthy niche in mice is a very encouraging observation. These results are yet to be translated into clinical use Clinical trials based on similar hypothesis are currently underway. We hope that the results of the present study may help gain insight into the underlying mechanisms that lead to infertility in cancer survivors and also help restore fertility in azoospermic adult survivors of childhood cancer who were denied the opportunity to cryopreserve gonadal tissue prior to therapy. Also this approach to restore fertility may be of use to patients in our setting in whom ART is not a viable option due to financial constraints. In azoospermic adult cancer survivors desirous of starting a family, autologus mesenchymal cells from bone marrow/adipose tissue could be transplanted in the testis with the hope of spermatogenesis restoration.

## Abbreviations

ACT clinic: After Completion of Therapy clinic
ES cells: Embryonic stem cells
iPS cells: Induced pluripotent stem cells
PGCs: Primordial germ cells
VSELs: Very small embryonic like stem cells
US FDA: United States Food and Drug Administration
